# Dosage compensation on the active X chromosome minimizes transcriptional noise of X-linked genes in mammals

**DOI:** 10.1186/gb-2009-10-7-r74

**Published:** 2009-07-13

**Authors:** Shanye Yin, Ping Wang, Wenjun Deng, Hancheng Zheng, Landian Hu, Laurence D Hurst, Xiangyin Kong

**Affiliations:** 1 The Key Laboratory of Stem Cell Biology, Institute of Health Sciences, Shanghai Institutes for Biological Sciences, Chinese Academy of Sciences/Shanghai JiaoTong University School of Medicine, South Chongqing Road, Shanghai 200025, PR China; 2 State Key Laboratory of Medical Genomics, Ruijin Hospital, Shanghai Jiaotong University, Rui Jin Road II, Shanghai 200025, PR China; 3 Department of Biology and Biochemistry, University of Bath, Bath, BA2 7AY, UK

## Abstract

**Background:**

Theory predicts that haploid-expressed genes should have noisier expression than comparable diploid-expressed ones with the same expression level. However, in mammals there are several classes of gene that are monoallelically expressed, including X-linked genes, imprinted genes and some other autosomal genes. Does it follow that the evolution of X chromosomes in eukaryotes comes at the cost of increased transcriptional noise in the heterogametic sex? Moreover, is escaping X-inactivation in mammalian females associated with an increase in transcriptional variation? To address these questions, we analyze gene expression variation between replicate samples of diverse mammalian cell lines in steady-state using microarray data.

**Results:**

We observe that transcriptional variation of X-linked genes is no different to that of autosomal genes both before and after control for transcript abundance. By contrast, autosomal genes subject to allelic exclusion do have unusually high noise levels even allowing for their low transcript abundance. The prior theory we suggest was insufficient, at least as regards X-chromosomes, as it failed to appreciate the regulatory complexity of gene expression, not least the effects of genomic neighborhood.

**Conclusions:**

These results suggest that high noise is not a necessary consequence of haploid expression and emphasize the primacy of expression level as a determinant of noise. The latter has consequences for understanding the etiology of haplo-insufficiency and the evolution of gene expression levels. Given the coupling between expression level and noise on the X-chromosome, we suggest that part of the selective advantage of dosage compensation is noise abatement of X-linked genes.

## Abstract 2

Comparison of gene expression variation in autosomal and X-linked genes reveals that high transcriptional noise is not a necessary consequence of haploid expression.

## Background

Apparent stochasticity, or 'noise', can be observed in many aspects of a biological system, ranging from loss of cell-cycle synchronization in an initially synchronized population of cells to different hair color of genetically identical cloned cats [1-5]. A potential source of phenotypic variability is the stochastic variation in gene expression, which influences most aspects of cellular behavior [3,5-7]. Transcriptional noise is known to play a crucial role in such heterogeneity. For any given mRNA or protein, this noise can be quantified by estimating the amount of variation in abundance between otherwise similar replicate cells or samples [8].

There are several environmental and genetic factors that could influence gene expression noise. As regards transcriptional noise, ploidy is thought to be one such determining factor. Using simulations to illustrate the influence of gene copy number on gene expression noise, Cook *et al*. [9] demonstrated that haploid expression should be noisier than diploid expression. This is for at least distinct two reasons. First, if haploid expression is associated with lower levels of the relevant product, then higher noise can result as noise and dosage can be negatively correlated [1,10], the effects of stochasticity being more profound when molecules are rarer. A negative correlation between noise and product abundance is indeed observed in yeast [8,10]. Second and more crucially, Cook *et al*. [9] argue that, even if mean dosage is compensated, haploid expression should still be noisier because haploid systems have a higher probability of interrupted gene expression than diploid systems; there is enhanced predictability of gene expression from integrating independent stochastic events permitted by having two copies producing the same product [9].

Differences in noise between haploid and diploid expressed genes have immediate relevance to the understanding of the causes of haplo-insufficieny [9]. Indeed, reduction in dose of a gene in a heterozygous knockout could increase noise both if dosage is reduced and owing to haploidy *per se*. If we suppose there to be some threshold level for proper functioning, then high noise associated with a reduction in dosage may well have phenotypic consequences. The same theory is also evolutionarily relevant if either too little or too much of the RNA or protein disrupt the functioning of cellular networks, so conferring a fitness cost [7,11,12]. *A priori*, assuming that at any given time there exists a unique optimal level of any molecule, we expect that selection should act to minimize transcriptional noise of most genes (one possible exception are genes whose products are necessary for response to environmental fluctuations, such as metabolic import channels or stress response [13]). The finding of low noise for essential genes [8,12] and for haplo-insufficient genes [14], even controlling for expression level, is consistent with such expectations, given that selection on dosage of essential and haplo-insufficient genes is, by definition, likely to be stronger than on non-essential genes.

In this context, expression from both parental alleles is beneficial for at least two reasons: firstly, owing to dominance, diploid organisms can mask the effects of deleterious recessive mutations; secondly, biallelic-expression guards against effects of dosage fluctuation. However, in mammals there are several classes of gene that are monoallelically expressed. These include X-linked genes, which by necessity are haploid when in males and most are also subject to X-inactivation in the somatic cells of females. There are also haploid-expressed autosomal genes. For example, imprinted genes are haploid-expressed in a parent-of-origin manner, while a further distinct class is the widespread monoallelically expressed autosomal genes (MAs) [15].

Given the postulate that haploid systems should be noisy systems, the evolution of heteromorphic sex chromosomes from a diploid-expressed ancestor is expected to come at the cost of increased noise in gene expression. However, one might suppose that just as dosage is compensated between autosomes and the X chromosome, so also noise is compensated. In part, noise compensation might result from dosage compensation, but the results of [9] proposed that, owing to haploidy, noise should still be high. To ask whether X-linked genes have high noise or fully compensated noise we compare their noise levels to diploid-expressed comparators. We start by verifying, theoretically and empirically, our noise metric.

## Results

### Noise can be measured employing replicate populations of cells

**Figure 1:**
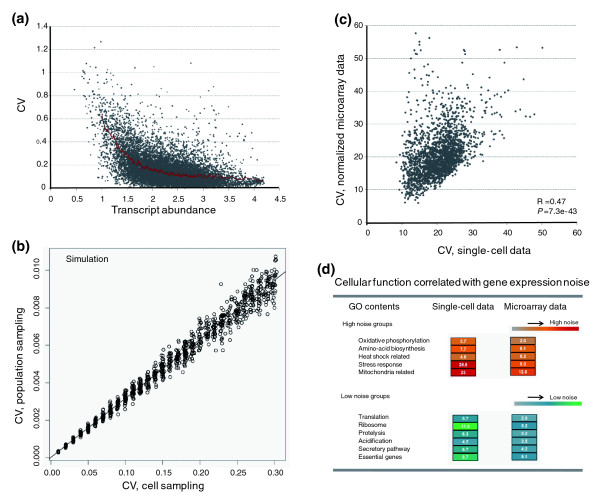
Measuring transcriptional noise employing microarray data. **(a) **Negative correlation between gene abundance and expression variation demonstrated by data from HaCAT cells cultured in the same normal condition. Each dot presents each gene while the red curve presents the mean expression variation in a running window of 100 genes. **(b) **Noise on the between-cell level and that on the between-population level are highly correlated according to simulation. In this simulation we considered a population of 10,000 cells all with the same underlying mean abundance and a given standard deviation. First, CV was calculated for 10,000 randomly generated data points (cell sampling). Next, we considered 100 populations of size 1,000 with the same mean and standard deviation. We simulated each using the same mean and standard deviation then considered the between-population CV as being the standard deviation between the Means of 100 populations/Mean of the means of the 100 populations. **(c) **Noise on the between-cell level and that on the between-population level are highly correlated, as demonstrated by experimental data in yeast. The plot shows the noise value (CV) measured by our microarray approach plotted against that measured by a previous single-cell approach. The noise values (CV) measured by our microarray are normalized to be comparable to the previous single-cell data in yeast (with equalized mean CV values). **(d) **Cellular function is correlated with transcriptional variation. For example, proteins participating in stress response exhibit large variation whereas proteins participating in translation exhibit low variation. The high or low variation groups identified by the single-cell approach and microarray data are highly consistent, indicating that microarray data can accurately identify high or low noise classes of gene. GO, Gene Ontology.

High resolution noise assays [8] have successfully compared the titer of a protein between single cells of a population in yeast. By contrast, in this study, we use microarray data from replicate populations of cells to evaluate transcriptional noise in mammalian cells. We thus define transcriptional noise as the coefficient of variation (CV; standard deviation/mean) of gene abundance assayed between replicates of populations of the same cell type under the same normal condition (Figure 1a). Our result is highly consistent with previous single-cell studies in yeast [8] such that the overall transcriptional variation is negatively associated with transcript abundance. The variation seen between replicate populations of the same cell types should also provide an unbiased estimation of noise. This is because if there is much variation between cells in a transcript's level, there should also be relatively large variation between replicate cell populations. To demonstrate this, we first performed a simulation in which we mimicked the two methods for assaying noise (on the between-cell and between-population levels), and found, as expected, a linear positive relationship for the noise assay between the two approaches (Figure 1b).

It has already been demonstrated that variation in gene expression measured by microarray data is highly consistent with single-cell data in yeast [8,10]; this is because genes sensitive to random fluctuations in the microenvironment or the activity of regulatory factors at the single-cell level are also sensitive to population-level perturbations in the microenvironment or the genetic makeup of regulators, with epigenetic mechanisms as the common denominator [16,17]. Indeed, we found a good correlation between noise values measured by our microarray approach and by the single-cell approach in yeast (Figure 1c), consistent with prior reports [16,17]. Moreover, prior single-cell data suggest that gene expression variation is related to gene function. Subgroups of genes that respond to environmental changes, for example, are considered to be 'noisy' whereas some others, like those involved in protein synthesis, are considered to be 'quiet' [8,10,14]. In comparing the gene expression variation in yeast measured by our *en masse *microarray approach with that of a previous single-cell study in about 2,000 genes with both microarray data and single-cell data available [8], we found that the results of these two approaches are highly consistent such that gene classes reported to be noisy at the between-cell level are also noisy at the between-population level (Figure 1d; significance of difference in noise level between each subgroup and all genes was determined by Mann-Whitney U-test). This benchmarking supports the sensitivity and reliability of our method to evaluate transcriptional variation with microarray data.

### Transcriptional noise is the same for X-linked genes and autosomal genes

**Figure 2:**
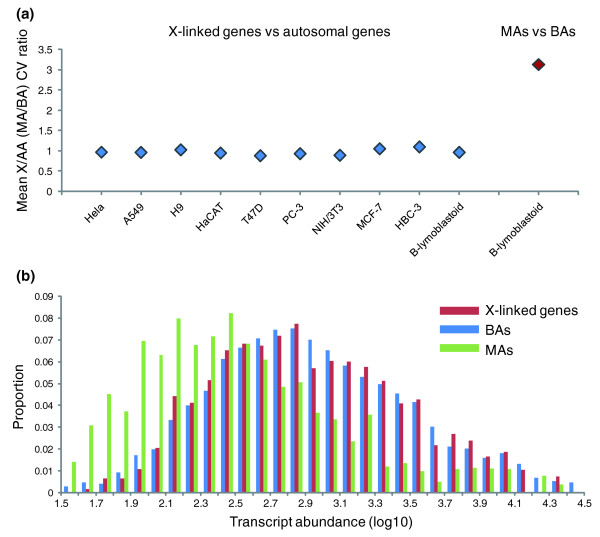
Transcriptional variation is balanced between X-linked genes and biallelically expressed autosomal genes (BAs), while the variation of monoallelically expressed autosomal genes (MAs) is higher than thatof BAs. **(a) **X chromosome:autosome (X/AA) mean transcriptional variation ratios were calculated for the mammalian cell lines noted at the bottom, and that of MAs versus BAs (MA/BA) was calculated for B-lympoblastoid, in which these MAs were identified. **(b) **Distribution histograms of BAs (blue bars), X-linked genes (red bars) and MAs (green-bars) at different gene expression levels with data from all the cell lines analyzed. X-linked genes and BAs are distributed symmetrically, while MAs are enriched in the low-expression regions.

To evaluate the effects of ploidy on gene expression variation, we considered genes on the X chromosome and autosomes. We also considered MAs and biallelically expressed autosomal genes (BAs) in human B-lymphoblastic cell lines (in which these MAs were identified [15]). As imprinted genes are relatively rare and even fewer are expressed simultaneously in the relevant cells, we excluded them from this study (Figure 2a).

In apparent contradiction of the prior theory [9], we find that the mean transcriptional variation of X-linked genes is no different to that of autosomal genes in any of the cell lines analyzed (mean CV of X = 0.149 ± 0.120; mean CV of autosomal genes = 0.151 ± 0.124; *P *> 0.05, Mann-Whitney U-test). By contrast, analysis of MAs found their mean variation value to be more than threefold higher than that of BAs, the difference being significant (mean CV of MAs = 0.457 ± 0.190; mean CV of BAs = 0.151 ± 0.124; *P *= 1.5E-7, Mann-Whitney U-test).

### Up-regulation of gene expression is a possible reason for the lower-than-expected transcriptional variation of X-linked genes

Why do X-linked genes have lower noise levels than MAs, although both are functionally haploid? One distinct difference is their gene expression levels. Transcript/protein abundance has been proposed as a determinant of between-gene variation in gene expression noise in yeast, such that genes with low abundance products are more likely to have high noise [8,10] (Figure 1a). Supporting the notion that abundance is the key determinant, our distribution histograms of gene expression levels in mammalian cells demonstrate that MAs are preferentially enriched in the low-expression class while X-linked genes have a range of expression values similar to that of BAs (Figure 2b). This largely concurs with the notion that the transcriptional output from the single-copy X chromosome is up-regulated to equal that of the average autosomal gene in mammals [18].

**Figure 3:**
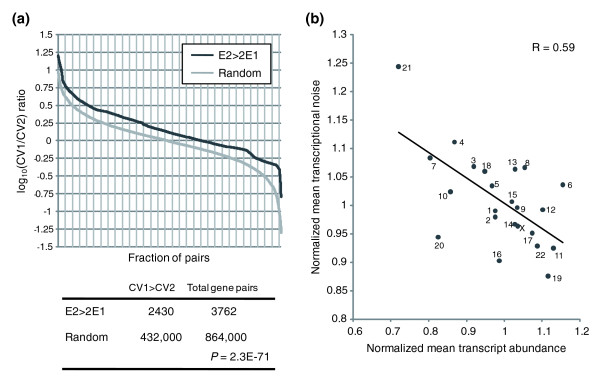
Up-regulation of X-linked gene expression possibly facilitates the lower-than-expected transcriptional variation. **(a) **Considering the gene expression level and transcriptional variation of the same gene in two different cell lines (of the same species), when the gene expression is twofold lower in one cell (E1) than another (E2), we calculated the noise ratio CV1/CV2 as one group (black curve after sorting and logarithmic transformation) and as random pairs (grey curve after sorting and logarithmic transformation). The number of different pairs is shown, which demonstrates that transcriptional variation is significantly reduced when gene expression is up-regulated. **(b) **Regression of the mean transcript abundance of each chromosome against the mean transcriptional noise of each chromosome. On a chromosomal level transcriptional noise is negatively correlated with gene abundance variation.

To demonstrate the effect of reducing transcriptional noise by up-regulating gene expression on a global scale, we considered genes that are more than twofold up-regulated in one cell line (E2 > 2E1). We then calculated the pair-wise ratio of transcriptional noise CV1/CV2, where CV1 is the transcription variation of the gene in the cell line in which it had lower mRNA abundance. Then we compared the CV1/CV2 ratios selected by this criterion with those of randomly selected pairs, regardless of differences in abundance of their transcripts between the cell lines. The probability of observing higher CV1 than CV2 in the E2 > 2E1 group is significantly higher than in the randomized group (Figure 3a; *P *= 2.3E-71, chi-square test). That transcriptional noise is negatively correlated with transcript abundance is also evident on the chromosomal scale: chromosomes with a relatively high mean gene expression level always have a relatively low mean transcriptional noise value and *vice versa *(Figure 3b).

### Monoallelically expressed genes still show high noise levels after controlling for their expression level, but X-linked genes do not

Above we have shown that the high transcriptional noise of MAs is due, in part, to their low expression levels, while the lower-than-expected noise of X-linked genes is, in large part, a consequence of their compensated expression levels. Given this, is there any evidence that haploid expression might be especially noisy, beyond any consequences of modified expression level? To determine this, we asked whether the transcriptional noise of MAs and X-linked genes is still high after controlling for expression level.

Employing data available from all human cell lines, we partitioned genes into 15 bins by expression level, so that all the genes in each of the 15 bins have approximately equal levels. Genes within each bin were then equally separated into three groups by their transcriptional noise level. We then analyzed whether MAs or X-linked genes are enriched in the group with the highest noise within each bin (that is, after controlling for gene abundance). A Fisher's exact test demonstrated that in none of the 15 bins are X-linked genes skewed towards high noise compared with autosomal genes (*P *> 0.05). However, after excluding five high gene abundance bins in which the number of MAs was insufficient for statistical analysis, seven out of ten bins exhibited significant enrichment of MAs in the high noise fractions compared to BAs (*P *< 0.05). Similar results were found when using 30 bins instead of 15, and when dividing each bin into two or four groups instead of three (data not shown). We conclude that X-linked genes have noise levels expected given their expression levels, while MAs appear to have noise levels greater than expected after controlling for expression level. In contrast to theoretical expectations [9], high noise is thus not a necessary consequence of haploid expression.

That X-linked genes have expected transcriptional noise levels given their dosage suggests that haploidy *per se *need not impact on noise. This indicates that any impact of ploidy on transcriptional noise might be overshadowed by the stochastic nature of other events in gene expression, such as transcription factor complex formation, RNA polymerase recruitment, and translational efficiency. What is unclear, however, is whether MAs have high noise, after controlling for expression rates, because they are haploid expressed or because they are the sort of genes that, *a priori*, would be expected to have high noise, such as stress response genes or more dispensable genes. A case can be made that this might indeed be the explanation. It is notable, for example, that the haploid X chromosome contributes much to sex determination and differentiation, and many genes on the mammalian X chromosome are involved in important biological processes, such as brain function and spermatozoa maturation [19-22]. Regulatory mechanisms that help to minimize noise may guarantee that downstream processes are not burdened by fluctuations in levels of the gene product. By contrast, many MAs with low expression levels (and high noise) are cytokines, antigen receptors and odorant receptors. From examination of the sorts of genes subject to monoallelic expression, a case can then be made that the high noise is expected. For such genes monoallelic expression is probably necessary for recognition specificity in the immune and nervous systems [15,23,24]. Importantly, such biological functions are controlled by the amount of cytokine-producing cells rather than the concentration of cytokine produced in each cell, so high transcriptional noise might not be a crucial concern. Moreover, diversity in the phenotypic states at the single-cell level might maximize the population's biological function and ability to cope with changing environmental challenges [3,5,7,25]. Given that transcriptional noise could be advantageous for such genes, we surmise that the present data are not adequate to establish whether haploidy *per se *ever leads by necessity to higher levels of expression noise even after controlling for expression level.

### Escaping X-inactivation does not lead to a measurable rise in transcriptional variation

The conclusion that X-linked genes have expected transcriptional noise levels given their expression levels is further verified by comparison with genes that escape inactivation. A comprehensive X-inactivation profile of the human X chromosome shows that, in total, about 15% of X-linked genes escape inactivation to some degree and an additional 10% show variable patterns of inactivation in descendant cells from the same origin, and are expressed to differing degrees than some 'inactive' X chromosomes [26]. These genes might potentially contribute to sexually dimorphic traits, to clinical symptoms linked with X chromosome abnormalities and, more importantly, to expression heterogeneity and phenotypic variability among females [26].

**Figure 4:**
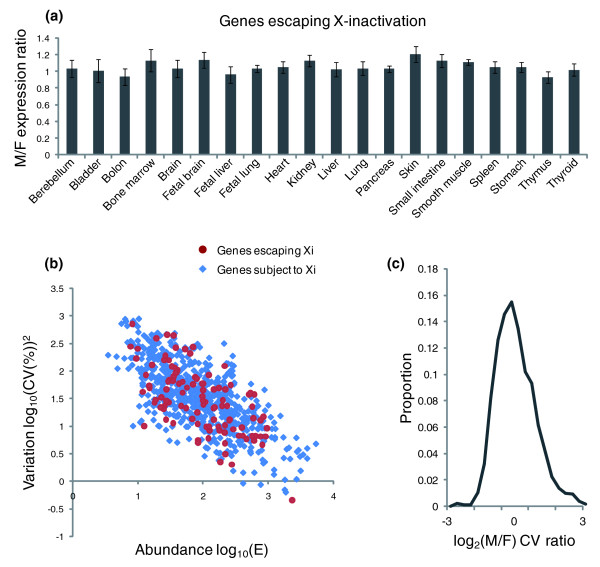
Escaping X-inactivation (Xi) causes no measurable increase in transcription level or noise. **(a) **Male/female (M/F) expression ratios (mean ± standard error of the mean) of each gene escaping Xi in 20 non-gender-specific tissues are shown. No increase in expression levels was observed in females. **(b) **Correlation of the noise values (log_10_(CV(%)^2^)) with gene expression values (log_10_(abundance)) of X chromosome genes subject to Xi (grey dots) and escaping Xi (black dots) in HeLa cells. No skewed enrichment in expression or fluctuation of genes escaping Xi was observed. **(c) **Distributions of logarithmic male/female (M/F) CV ratios of genes escaping Xi; the M/F ratios are close to 1 in most cases.

Genes escaping X-inactivation have similar expression levels to those that are haploid expressed (*P *> 0.05, Student's paired two sample *t*-test) and male-to-female (M:F) expression ratios of these genes were close to 1 in all the non-gender-specific tissues (Figure 4a). Moreover, genes that escape X inactivation do not show greater transcript abundance or transcriptional noise in comparison with other X-linked genes, as demonstrated by data from HeLa cells (Figure 4b). As we would then expect, there is no observable difference in transcriptional noise between genes escaping X inactivation and genes subject to X inactivation in any of the female cell lines used (*P *> 0.05, Mann-Whitney U-test). M:F transcriptional noise ratios of each gene escaping X inactivation in randomly paired male-female cell lines approximately follow a normal distribution, with most values around 1 (Figure 4c). No measurable differences in transcriptional noise levels of these genes were observed between male and female cell lines (*P *> 0.05, Student's paired two sample *t*-test). These results indicate that escaping X inactivation does not necessarily affect transcriptional noise as expression levels are, on average, the same. These results emphasize the primacy of dosage, over haploid expression, in the determination of transcriptional noise level.

## Discussion

Owing to monosomy, X chromosome gene products would face both the potential problems of dosage deficiency and high gene expression noise. This requirement for dosage balance has led to a dosage regulating mechanism that restores equivalent gene expression levels between haploid expressed X chromosomal genes and diploid autosomal ones. In this study, we further suggest that, to some major degree, the evolution of higher expression rates from X-linked genes also reduces the transcriptional variation (noise). Indeed, we find no evidence that, controlling for expression level, haploidy comes at any cost, as regards noise level, for genes on the X-chromosome. Dosage compensation is hence also full noise compensation. Our results support the view that haploidy *per se *need not have a detectable effect on noise and emphasize the pre-eminent importance of dosage in noise variation.

While much work examines how dosage compensation is achieved (for example, [18,27,28]), why the transcriptional level of dosage deficient X-linked genes is fine-tuned to equal that of autosomal genes is less well resolved. Our findings have provided a potential further explanation for why X chromosome dosage compensation is established, not just for dosage balance but also for minimization of potentially deleterious noise of X-linked genes.

These findings promote further questions. First, why is haploidy *per se *apparently irrelevant on X chromosomes (but not necessarily on autosomes), when Cook *et al*.'s model predicted otherwise? Second, is the coupling of noise and dosage necessarily a direct coupling as we presume and, if so, what are the broader implications of the pre-eminence of dosage in the determination of noise levels?

### Why might haploidy be irrelevant on X-chromosomes?

Why could we not detect any of the inherent stochasticity associated with haploidy predicted by [9] when looking at X-linked genes, while such an effect could not be excluded for MAs? We hypothesize that this may be a consequence of Cook *et al*.'s theoretical treatment of each gene in isolation, ignoring the genomic context. In mammals, for X-linked genes, all the activity is concentrated on one chromosome. Consequently, on the active X chromosome, gene expression is up-regulated and chromatin structure is more likely to be in the open form. This may act to reduce noise levels below those expected for autosomal genes with the same net output, especially if the keeping of chromatin open is reinforced by the activity of flanking genes. It is well known that adjacent genes tend to be co-expressed probably, in part, because they share the same chromatin environment, and the transcriptional status of one gene likely affects other genes in the vicinity [14,29,30].

Several models are consistent with such a notion. For example, the binding of a transcription factor to one gene opens the chromatin structure such that the neighboring gene could have increased accessibility to transcription factors to start its own transcription. Similarly, if one gene is being transcribed, then the focal gene would have less chance to close its chromatin structure. If genes in the neighborhood are all in steady state, then the focal gene would also be affected by this genomic atmosphere.

This suggests the possibility that transcriptional variation of a focal gene could be modified passively by genes in the vicinity. Random activation and inactivation of the gene promoter, resulting from changes in chromatin structure or from the stochastic binding and unbinding of transcription factors, may be determinant contributors to transcriptional noise [6,14]. Put differently, part of the stochasticity of gene expression derives from stochastic failure of transcription factors to 'find' the gene promoter. If chromatin is more often open, this stochastic element is reduced. Some previous studies support this idea. Notably, in an SWI6 repositioning experiment, after changing the chromosomal position of PSWI6 by integrating it from the ade2 locus, with high transcriptional noise, to the his3 locus, with a low level of transcriptional variation, its variation at his3 was substantially reduced [6]. It has also been reported that expression noise is influenced by the density of essential genes in the chromosomal vicinity, independent of protein abundance. Domains with a high density of essential genes with low levels of transcriptional noise harbor more phenotypically important nonessential genes, these being those that would benefit from the low noise environment [14] that corresponds to open chromatin.

If such an effect were to explain part of the low noise of X-linked genes, we expect to see a correlation in the level of transcriptional variation between adjacent gene pairs. To address this, we calculated the metric:



- where CV_i _is the variation in gene expression associated with gene i and CV_j _is the variation in gene expression associated with its adjacent gene j. The resulting distribution of d_ij _for about 6,000 adjacent gene pairs was compared to the distribution of 6,000,000 randomized gene pairs in each of the human cell lines tested here. We find that the deviation between adjacent gene pairs (d = 0.259 ± 0.196 (mean ± standard error of the mean)) is smaller than for random gene pairs (d = 0.378 ± 0.233) (*P *= 3.2E-6, Mann-Whitney U-test). We get similar results when doing the comparison separately for each cell type (data not shown). The above evidence indicates that fluctuations in transcription of adjacent genes are tightly associated, which might be partly explained by sharing the same active/inactive status of the loci. This aspect was missing in Cook's models of haplo-insufficiency in which the transcriptional/noise environment of each gene was considered in isolation. It may be relevant that MAs do not cluster [15] and are not up-regulated.

### Implications of the pre-eminence of dosage in noise determination

Above we presume that increases in dosage are likely to cause *de facto *decreases in noise. Our data, however, are consistent with, but not evidence for, such a coupling, being largely correlation based. It could be that genes with high dosage are subsequently selected to have low noise, or that genes with intrinsically low noise are more likely to evolve higher expression levels. The presumption that noise and dosage are mechanistically coupled is, however, consistent with both models of noise creation [1,10] and experimental evidence showing that the mean protein titer derived from transgenes across different conditions negatively correlates with the noise level [1]. Likewise, insertion of a transgene to a genomic domain in which it has higher expression levels causes a reduction in noise levels [6]. Our finding of a difference in noise of the same gene when highly and lowly expressed provides further support. Given these results, it seems reasonable to presume that the negative correlation that we observe is owing to a direct mechanistic coupling.

If the coupling is indeed direct and as profound an influence on noise levels as our results would suggest, then the effects of mean dosage *per se *cannot be easily isolated from the resulting effects on noise. Our results thus have bearing for both the likely etiology of haplo-insufficiency and the evolution of expression rates.

As regards haplo-insufficiency, Cook *et al*. [9] proposed that even if dosage is unaffected, haploid expression *per se *should lead to higher noise. Our results suggest that this is not such an important effect. While we cannot definitively rule this possibility out, by far the greater effect on noise would be mediated by a reduction in mean dosage, this being coupled with an increase in noise. Even if a cell was viable if the mean half dose were stably maintained, the increase in noise may ensure that protein dosage occasionally falls too far and cell lethality ensues.

The primacy of dosage in the determination of noise may, in addition, be important to the evolution of expression rates and explain some of the between-gene variation in expression rates [31]. Essential genes, by definition, are those for which a reduction in dosage below some threshold is immediately and severely deleterious (that is, lethal). Let us suppose that with no noise (that is, in a deterministic model) there exists an optimal level of gene expression. With noise, however, at this optimum mean level, dose can fall below the lethality threshold. One way to minimize the chances of this would be to increase expression levels beyond the optimal mean level. By modifying dose and noise concomitantly, the evolution of higher than 'optimal' expression levels greatly minimizes the chances that fluctuations in dose would ever go below the lethality threshold. This is not just because the mean dosage is further from the threshold, but in addition the fluctuation in levels is lower too. Others have gone further to suggest that it is noise alone that is the focus of selection on essential genes [31], but the move away of the mean level from the lethality threshold seems to us an inevitability of any such selection. In this view, the fact that essential genes have high expression levels [32] may be because being essential, high levels of expression are selectively favorable owing to a coupling of noise and dosage [31]. This noise-modification view of expression levels is consistent [12] with the otherwise counter-intuitive finding that mRNA from essential genes has a short half-life [33], this being a mechanism to reduce noise. The alternative, more classical view would be to suppose that expression level is determined by the deterministic optima and that genes expressed at high levels are more likely to induce large fitness effects when their abundant product is absent.

The noise-dosage correlation may be relevant to the problem of the successful invasion of duplicate genes and the selective forces operating on gene loss events following whole genome duplication. We leave any such consideration to further analysis. On a broader scale it is tempting to suggest that the correlation may be of importance for the evolution of ploidy and for the fate of whole genome duplications. We caution, however, that extrapolation of results from X chromosomes to these issues is non-trivial, not least because noise levels are also expected to vary with absolute cell dimensions.

## Conclusions

In this study, we reveal that, to some major degree, the evolution of higher expression rates from X-linked genes also reduces the transcriptional variation (noise). Indeed, we find no evidence that, controlling for expression level, haploidy comes at any cost, as regards noise level, for genes on the X-chromosome. X chromosome dosage compensation is hence also full noise compensation. These results suggest that haploidy *per se *need not result in higher transcriptional noise as a prior model claimed. These results emphasize the primacy of expression level as a determinant of noise. Such dosage-noise covariance has significant importance for understanding the etiology of haplo-insufficiency and the evolution of gene expression. For example, our results are consistent with the possibility that the high expression level of essential genes may have been selected as it both increases the distance between mean dosage and lethal threshold levels and reduces noise. Our findings add to the usual supposition that dosage compensation is necessary to balance abundance of gene products, additionally noting that, commensurate with such dosage modification, will be noise minimization for X-linked genes. Assuming noise to have selective consequences, this is likely to be a previously unrecognized component of any selection for dosage compensation of the active X chromosome.

## Materials and methods

### Data sources

Gene expression profiles were obtained from National Center for Biotechnology Information (NCBI) Gene Expression Omnibus [34] and European Bioinformatics Institute ArrayExpress [35]. To eliminate the influence of different platforms, only data generated with the Affymetrix Human Genome U133 plus 2.0 Array and Mouse Genome 430 2.0 Array were used in our study, along with the Yeast genome 2.0 Array. All together, 80 expression profiles for yeast, and 720 expression profiles from 9 human and mouse cell lines were analyzed. Genes escaping X-inactivation were obtained from [26] while the list of MAs was obtained from [15].

### Microarray data processing

Microarray raw data files were processed using the GeneSpring software based on the annotation files available at the Affymetrix website. Data were extracted in CEL file format, and reanalyzed using GeneSpring. Individual arrays were assessed for various quality control parameters as described in the Affymetrix GeneChip Expression Analysis technical manual. All subsequent analysis was conducted in GeneSpring GX (version 7.2; Agilent Technologies) and Excel 2000 (Microsoft Corp., Redmond, WA, USA). Probes were excluded from further calculations if their background-corrected intensities were below zero and/or if spots were flagged as non-uniformity outliers as determined by the image analysis software. After elimination of background, the mean fluorescence intensity of duplicated spots representing the same gene was calculated and normalized to the mean fluorescence intensity of the whole array for all arrays of the same cell.

From each set of arrays extracted from the databases, a gene expression distribution histogram (Microsoft Excel) was created to determine whether expression values (log_2 _based and binned) for all genes surveyed followed a normal distribution. After precluding the unexpressed genes based on the signal intensities of perfect match (PM) and mismatch (MM) probes of microarrays, the percentage of X-linked genes expressed is about 4% of the total numbers of genes, consistent with the percentage of total X-linked genes in the mammalian genome, indicating that no more X-linked unexpressed or extremely low expressed genes were precluded from our analysis.

### Gene Ontology and annotation information

Gene annotation information was obtained from the Affymetrix website [36]. Organizations of Gene Ontology terms were established with DAVID 2008 [37].

### Correlation of gene expression noise between adjacent gene pairs

The physical maps of the transcripts were drawn using the assembly from the UCSC genome browser [38]. For each gene, the neighboring gene with the smallest chromosomal distance was identified, and the effect of gene proximity on expression noise was tested.

## Abbreviations

BA: biallelically expressed autosomal gene; CV: coefficient of variation; MA: monoallelically expressed autosomal gene; M:F: male-to-female.

## Authors' contributions

SY, XK, and LDH conceived and designed the experiments, and SY, XK, LDH, PW, WJ and LH analyzed the data. SY, LDH and XK wrote the paper.
